# Full life cycle changes of low impacted mandibular third molar associated cystic lesions and adjacent tooth root resorption: a retrospective study

**DOI:** 10.1186/s12903-024-04248-z

**Published:** 2024-05-02

**Authors:** Jiankang Zhang, Kun Zhang, Xueer Zhou, Li Ye, Yuanyuan Liu, Yiran Peng, Jian Pan

**Affiliations:** 1 State Key Laboratory of Oral Diseases & National Center for Stomatology & National Clinical Research Center for Oral Diseases & Department of Oral and Maxillofacial Surgery, West China Hospital of Stomatology, Sichuan University, No. 14, 3rd section of Renmin South Road, Chengdu, Sichuan 610041 China; 2 State Key Laboratory of Oral Diseases & National Center for Stomatology & National Clinical Research Center for Oral Diseases, Department of Oral Radiology, West China Hospital of Stomatology, Sichuan University, Chengdu, Sichuan 610041 China; 3 State Key Laboratory of Oral Diseases & National Center for Stomatology & National Clinical Research Center for Oral Diseases, Department of Pediatric Dentistry, West China Hospital of Stomatology, Sichuan University, Chengdu, Sichuan China

**Keywords:** Low impacted mandibular third molars, Adjacent tooth root resorption, Cystic lesion, Risk factor, Full life cycle

## Abstract

**Objective:**

Low impacted third molars are usually asymptomatic and are often found by X-ray examination. The removal of asymptomatic low impacted third molars is one of the most controversial clinical issues in oral and maxillofacial surgery.

**Methods:**

In this study, 806 patients with low impacted mandibular third molars (LIMTMs) (full bony impaction) were analyzed to determine the prevalence and risk factors for cystic lesions and adjacent tooth root resorption throughout the patients’ entire life cycle.

**Results:**

The results showed that the prevalence of adjacent tooth root resorption and cystic lesions was age-related, exhibiting a trend of first increasing and then decreasing; prevalence peaked at the age of 41 to 45 years old, the prevalence rates were 12.50% and 11.11% respectively. And the lowest prevalence rate was 2.86% and 2.44% in ≥ 61 group and 56- to 60-year age group respectively. Age was an independent risk factor for adjacent tooth root resorption of LIMTMs, whereas age and impaction type (especially inverted impaction) were independent risk factors for cystic lesions.

**Conclusions:**

The full life cycle management strategy for LIMTMs may need to be individualized. Surgical removal is recommended for LIMTMs in patients younger than 41 to 45 years, especially for inverted, mesioangular, and horizontally impacted LIMTMs. LIMTMs in patients older than 41 to 45 years may be treated conservatively with regular follow-up, but surgical removal of inverted impacted LIMTMs is still recommended to avoid cyst formation.

## Introduction

Impacted teeth are those that can only partially erupt or completely impacted because of obstruction by adjacent teeth, bone, or soft tissue, and they will not fully erupt in the future [1]. The third molar, located distal to the second molar, is the last tooth to erupt in humans. It is often impacted because of insufficient eruption space and is the most common type of impacted tooth. An impacted third molar may cause pericoronitis, caries, odontogenic cysts, tumors, damage to adjacent teeth, and crowding of anterior teeth. It may also cause periodontal problems associated with the adjacent second molar and even external root resorption [2–4]. According to the positional relationship between the impacted third molar and the occlusal plane of the second molar, third molar impaction can be categorized as high level, medium level, and low level [5]. The difficulty of extracting the impacted third molar increases with the depth of embedment [6].

Low impacted mandibular third molars (LIMTMs) generally have no obvious symptoms and are often found during imaging examinations [7, 8]. In many cases, however, by the time symptoms appear in the impacted mandibular third molars, irreversible damage such as adjacent tooth root resorption and jawbone cysts or tumors may have already occurred. Root resorption of the mandibular second molar is caused by contact between the second molar and the impacted third molar. The pressure exerted by the impacted third molar may lead to root resorption of the adjacent second molar. This pressure causes inflammation and resorption of the mandibular second molar through the action of osteoclasts [9]. Mesio-angulated and deeply impacted third molars were identified as risk factors for both root resorption in maxillary and mandibular second molars. Besides, age over 25 increased the risk of root resorption in second molars. For maxillary second molars, root resorption mostly occurred at the apical third, while the mandibular second molars root resorption was most frequently detected at the cervical third. Considering the presence of root resorption is associated with impaction type of third molars, watchful monitoring or prophylactic removal of impacted third molars should be deliberated especially for the patients over 25 years and with mesially inclined and deeply positioned third molars [10]. Nunn et al. [2] found that a “bony” impacted third molar increased the risk of incident second molar pathology by 2.16 (95% confidence interval [CI], 1.56–2.99). Oenning et al. [11] found that the incidence of root resorption in the adjacent second molars of the lower mesial and horizontal impacted mandibular third molars was 33.9% (19/56), and Fernanda et al. [12]found through a systematic evaluation that the prevalence of odontogenic cysts and tumors associated with impacted third molars was 5.3%. And the incidence of odontogenic cysts was 4.4%, the incidence of odontogenic tumors was 0.5%, the incidence of dentigerousc cysts was 2.1%, the incidence of odontogenic keratocysts was 0.5%, and the incidence of radicular cysts was 4.7%. Moreover, Ectopic mandibular third molar position could also lead to eruption impairment of the adjacent second molar. And the third molars related dentigerous cyst and tumor may interfere with the obstructed eruption path of the mandibular second molar. And the removal of third molars and the related bone lesion are conducive to the eruption of the impacted second molars [13].

Extraction of LIMTMs is more difficult than extraction of ordinary impacted third molars. Additionally, the surgical time is longer, the incision and wound are larger, and the affected area has a larger amount of bleeding and is slower to heal. The oral cavity is a bacteria-rich environment, and the incidence rate of postoperative complications is higher after LIMTM extraction than that of after other tooth extractions [14, 15]. Common intraoperative and postoperative complications include mandibular fractures [16, 17], tooth tissue displacement, nerve damage, infection, dry socket syndrome, facial swelling, and bleeding [18, 19]. Tooth tissue displacement is a common complication after extraction of low impacted third molars. It is often associated with broken roots of mandibular third molars, lingual displacement of the entire mandibular third molar into the sublingual and parapharyngeal spaces, or entry of the maxillary third molar into the temporal space. Postoperative neurological dysfunction is a common complication after extraction of low impacted third molars and is mainly characterized by dysfunction of the inferior alveolar and lingual nerves [20–23]. However, despite these the surgical risks and complications, the vast majority of patients who underwent a surgery of mandibular third molars extraction, make a good recovery. According to a prospective European multi-center study, treatment satisfaction and willingness to undergo similar surgery were reported by 92% and 95%, although 21% reported that the surgery and postoperative period had been worse than expected. Mean days with pain, sick leave, and swelling were 3.6, 2.1, and 3.6, respectively. Preoperative symptoms, dental anxiety level, and prolonged surgical time were associated with increased pain and swelling. Pell and Gregory classification (I-IIIC) were associated with impaired sensation of the lower lip and chin [24].

The removal of symptomatic low impacted third molars has been widely accepted, but the clinical decision-making process regarding asymptomatic low impacted third molars is one of the most controversial clinical issues in oral and maxillofacial surgery. The debate is focused on the fact that some asymptomatic low impacted third molars can lead to adverse consequences (such as adjacent tooth root resorption and cystic lesions), whereas other asymptomatic impacted third molars may remain clinically silent for life. The key to resolving this issue is to distinguish high-risk low impacted third molars, which would provide a valuable reference for clinical decision-making regarding LIMTMs.

This cross-sectional study based on cone-beam computed tomography (CBCT) images was performed to analyze the prevalence of root resorption and cystic lesions in teeth adjacent to LIMTMs throughout the entire life cycle; evaluate the correlation of these complications with age, gender, and impaction type; and further analyze the risk factors for adjacent tooth root resorption and cystic lesions related to LIMTMs. The null hypothesis of the study was that the prevalence of the adjacent root resorption and cystic lesions was independent of patient age, gender, and impaction type of LIMTMs. We anticipate that the findings of this study will serve as a reference for the full life cycle management of LIMTMs.

## Materials and methods

### Data collection

**Fig. 1:**
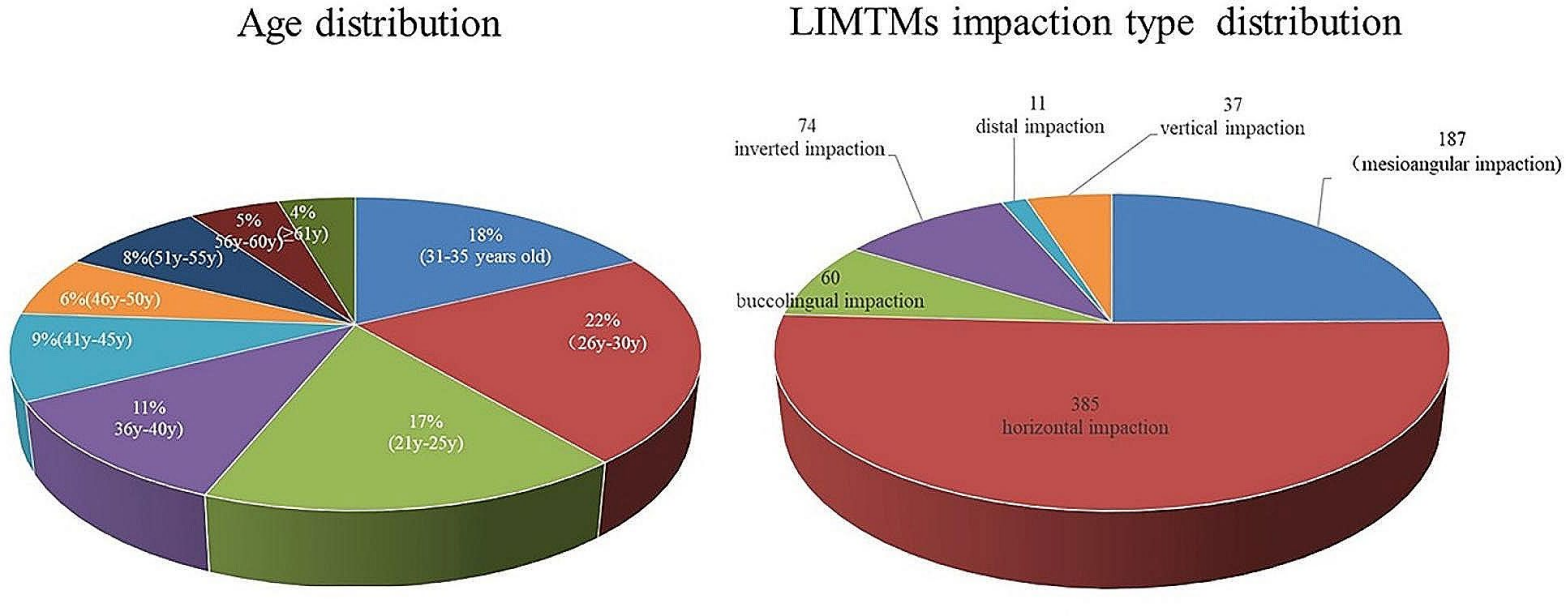
Age division characteristics and LIMTMs impaction type distribution of included cases

We collected the data of 806 patients among those seeking routine dental care from May 2023 to September 2023 at the West China Hospital of Stomatology, Sichuan University. The study was approved by the West China Hospital of Stomatology Institutional Review Board in May 2019 (approval number: WCHSIRB-D-2019-095) and performed in accordance with the Declaration of Helsinki and its guidelines. The inclusion and exclusion criteria of the study have been outlined in Table 1. Age division characteristics and LIMTMs impaction type distribution of included cases was showed in Fig. 1.

**Table 1:** Inclusion and exclusion criteria

Inclusion criteria	Exclusion criteria
(a) The age of all patients ≥ 21 years old.	(a) The roots of the third molar were less than two-thirds developed.
(b) With at least one low impacted mandibular third molar.	(b) The local anatomy and structure of the third and second molars were concealed on imaging examination because of high-density materials or other reasons.
(c) Having CBCT imaging data of the low impacted mandibular third molar and the adjacent second molar.	(c) The adjacent second molar was missing.
	(d) The mandibular second molar was affected by related diseases (e.g., tooth trauma, chronic periodontitis, or root canal treatment).

### Determination of external root resorption of adjacent second molars

The diagnostic criterion for external root resorption of adjacent molars on CBCT imaging was a broken, disrupted tooth root contour showing irregular erosion with low density. Root absorption was divided into three categories: mild, moderate, and severe absorption. In cases of mild resorption, the root absorption did not exceed half of the thickness of the root canal wall. In cases of moderate resorption, the root resorption exceeded half of the thickness of the root canal wall and did not involve the root canal system. In cases of severe resorption, the root resorption involved the root canal system.

### Prevalence of adjacent tooth root resorption in patients with LIMTMs

Based on the CBCT images, the prevalence of adjacent second molar root resorption was calculated for each age group: 21–25, 26–30, 31–35, 36–40, 41–45, 46–50, 51–55, 56–60, and ≥ 61 years. The degree of adjacent tooth root resorption was further analyzed in each age group, and the trend of the change in the prevalence of adjacent teeth root resorption with age was summarized.

### Risk factors of adjacent tooth root resorption

#### Effect of LIMTM impaction type

According to the angle formed between the long axes of the second molar and LIMTM, the LIMTM impaction type was categorized as follows: vertical impaction, − 10° to 10°; mesioangular impaction, 11° to 84°; horizontal impaction, 85° to 95°; distal impaction, − 11° to − 79°; inverted impaction, 95° to − 80°; and buccolingual impaction, not applicable. The relationship between the prevalence of adjacent second molar root resorption and the type of LIMTM impaction was analyzed and compared among the different age groups using the chi-square test. The horizontal impaction was set as control group, and other impaction types were set as variables.

### Effect of age and gender

The relationship between the prevalence of adjacent second molar root resorption and age and gender of patients was analyzed using Logistic regression, and then analyzing the risk factors for root resorption in teeth adjacent to the LIMTM. The risk factor of age was set as a continuous variable, and female was set as control group for risk factor of gender.

### Prevalence of cystic lesions in patients with LIMTMs

Based on the CBCT images, the prevalence of cystic lesions was calculated for each age group as listed above. The range of cystic lesions, positional relationship with the LIMTM, and invasiveness were further analyzed in each age group, and the trend of the change in the prevalence of cystic lesions with age was summarized.

### Risk factors of LIMTM related cystic lesions

#### Effect of LIMTM impaction type

According to the LIMTM impaction type as previous description, the relationship between the prevalence of cystic lesions and the type of LIMTM impaction was analyzed and compared among the different age groups using the chi-square test. The horizontal impaction was set as control group, and other impaction types were set as variables.

#### Effect of age and gender

The relationship between the prevalence of cystic lesions and age and gender of patients was analyzed using Logistic regression, and then analyzing the risk factors for LIMTMT related cystic lesions. The risk factor of age was set as a continuous variable, and female was set as control group for risk factor of gender.

### Statistical analysis

All data were analyzed using SPSS 22.0 statistical software (SPSS version 22.0, Chicago, IL). The relationship of the prevalence of adjacent tooth root resorption, cystic lesions, and the type of LIMTM impaction was analyzed and compared among the different age groups using the chi-square test. Logistic regression was used to analyze the risk factors for adjacent tooth root resorption and cystic lesions. Statistical significance was set at 0.05.

## Results

**Fig. 2:**
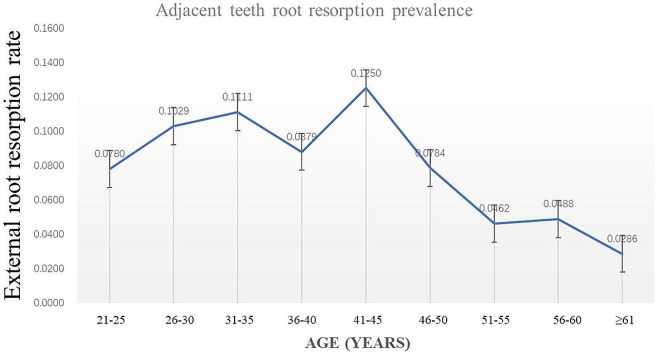
Changing trend of LIMTMs adjacent teeth root resorption prevalence with age. The prevalence of root resorption in the tooth adjacent to the LIMTM was age-related and showed a trend of first increasing and then decreasing; the prevalence peaked at the age of 41 to 45 years

In total, 806 patients with LIMTMs were included in this study, and 71 cases of adjacent tooth root resorption occurred (prevalence rate of 8.81%). The prevalence of root resorption in the tooth adjacent to the LIMTM was age-related and showed a trend of first increasing and then decreasing; the prevalence peaked at the age of 41 to 45 years (Fig. 2). Before the age of 45 years, the prevalence rate of adjacent root resorption of LIMTMs generally showed a gradually increasing trend. The lowest prevalence rate was 7.80% in the 21- to 25-year age group, the highest prevalence rate was 12.50% in the 41- to 45-year age group, and the prevalence rate showed a sharp decline after the age of 45 years. The prevalence of adjacent tooth root resorption was lowest in the ≥ 61-year age group (2.86%). The root resorption of adjacent teeth caused by various types of LIMTMs before the age of 45 years was severe in most cases, and that after the age of 45 years was mild and moderate in most cases.

### Relationship between age and prevalence of cystic lesions in patients with LIMTMs

**Fig. 3:**
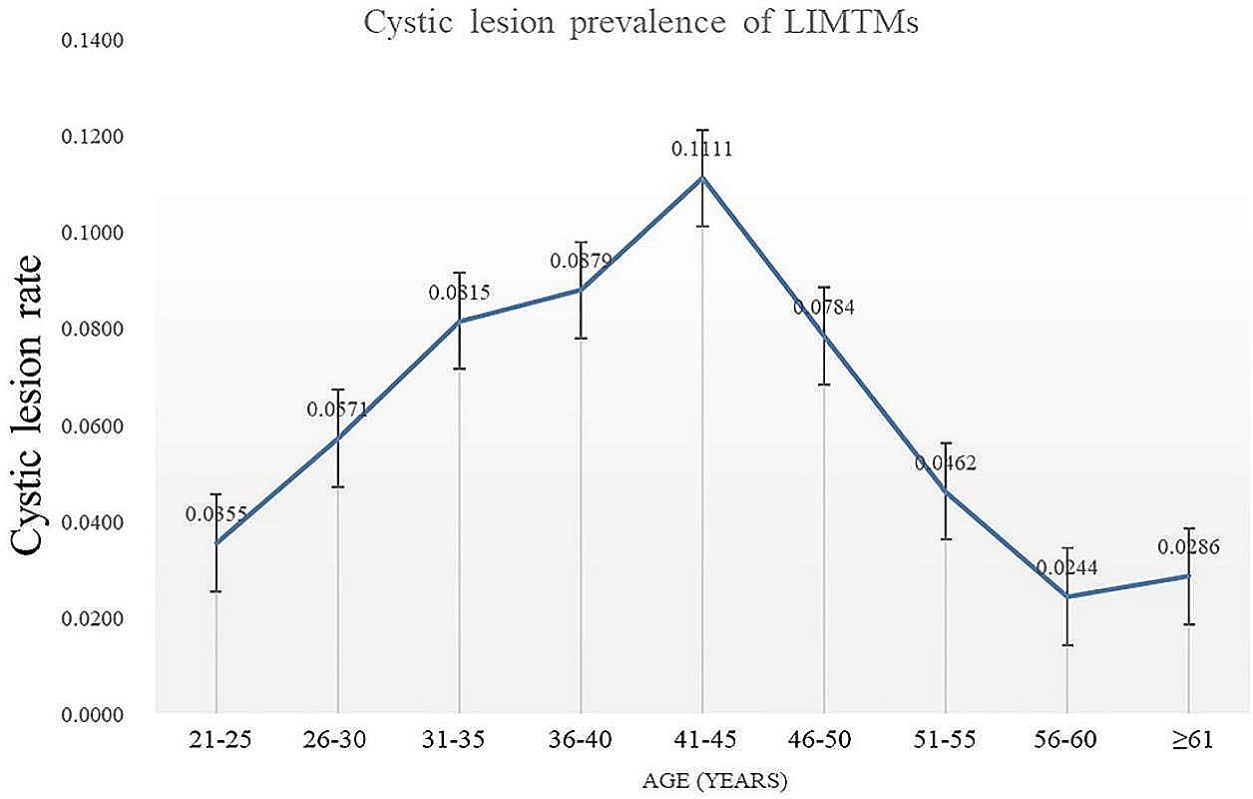
Changing trend of LIMTMs related cystic lesion prevalence with age. The prevalence of cystic lesions showed a trend of first increasing and then decreasing, and the prevalence peaked at the age of 41 to 45 years

**Fig. 4:**
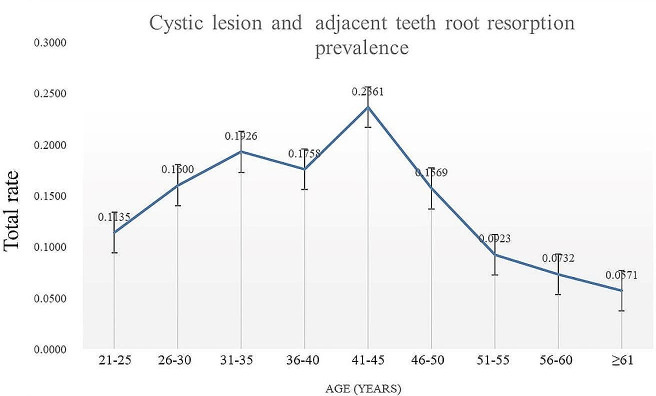
Changing trend of LIMTMs adjacent teeth root resorption and LIMTMs related cystic lesion prevalence with age. The changing trend of total prevalence of LIMTMs adjacent teeth root resorption and LIMTMs related cystic lesion with age was same as LIMTMs adjacent teeth root resorption

Among the 806 patients with LIMTMs in this study, and 51 developed cystic lesions (prevalence rate of 6.33%). The prevalence of cystic lesions showed a trend of first increasing and then decreasing, and the prevalence peaked at the age of 41 to 45 years (Fig. 3). Before the age of 45 years, the prevalence rate of cystic lesions of LIMTMs showed a gradually increasing trend. The lowest prevalence rate was 3.55% in the 21- to 25-year age group, the highest prevalence rate was 11.11% in the 41- to 45-year age group, and the prevalence rate showed a sharp decline after the age of 45 years. The prevalence of proximal root resorption of LIMTMs was lowest in the 56- to 60-year age group (2.44%). The changing trend of total prevalence of LIMTMs adjacent teeth root resorption and LIMTMs related cystic lesion with age was same as LIMTMs adjacent teeth root resorption and cystic lesion (Fig. 4).

### Effect of type of LIMTM impaction on incidence of adjacent tooth root resorption

We applied the chi-square test to analyze the CBCT data of all 806 patients with LIMTMs and found that inverted impacted LIMTMs had the highest rate of adjacent tooth root resorption (12.16%), while vertically impacted LIMTMs had the lowest rate (2.70%). The chi-square analysis results showed that the type of LIMTM impaction did not have a statistically significant influence on root resorption of adjacent teeth. We then further analyzed the relationship between the type of LIMTM impaction and adjacent tooth root resorption in each age group. The type of LIMTM impaction had a statistically significant effect on adjacent tooth root resorption in the 21- to 25-year age group but no significant effect in the other age groups (Table 2).

**Table 2:** Chi-square test for the effect of impaction types on root resorption of adjacent teeth

Age	Root resorption	Impaction types						Total	c^2^	*p*
		Vertical	Mesioangular	Horizontal	Distal	Inverted	Buccolingual			
21–25	Yes	0	4	3	0	2	2	11	26.227	0.0143*
	No	7	50	61	1	0	11	130		
26–30	Yes	1	7	8	1	0	1	18	2.4888	0.5641
	No	11	47	87	3	4	5	157		
31–35	Yes	0	2	12	0	1	0	15	4.2907	0.6253
	No	1	29	64	3	14	9	120		
6–40	Yes	0	2	6	0	0	0	8	3.2757	0.8412
	No	4	18	41	1	9	10	83		
41–45	Yes	0	1	3	0	3	2	9	5.5354	0.198
	No	3	12	35	0	9	4	63		
46–50	Yes	0	0	1	0	0	1	2	3.5482	0.5082
	No	4	9	21	1	9	5	49		
51–55	Yes	0	2	0	0	1	0	3	6.1156	0.1258
	No	2	11	35	0	9	5	62		
56–60	Yes	0	0	0	0	1	0	1	6.1156	0.1258
	No	2	7	21	1	6	3	40		
≥ 61	Yes	0	0	0	0	1	0	1	4.9755	0.4286
	No	2	4	20	1	5	2	34		
Total		37	205	418	12	74	60	806	3.2846	0.6562

* *p* < 0.05;** *p* < 0.01

### Effect of type of LIMTM impaction on incidence of cystic lesions

We applied the chi-square test to analyze the CBCT data of all 806 patients with LIMTMs and found that inverted impacted LIMTMs had the highest incidence of cystic lesions (33.78%), while mesioangular impacted LIMTMs had the lowest incidence of cystic lesions (2.93%). The chi-square analysis results showed that the type of LIMTM impaction had a statistically significant effect on the occurrence of cystic lesions. We then analyzed the relationship between the type of LIMTM impaction and the occurrence of cystic lesions in all age groups. The type of LIMTM impaction had a statistically significant effect on the occurrence of cystic lesions in the 26- to 30-year, 31- to 35- year, and 36- to 40-year age groups but no significant effect in the other age groups (Table 3).

**Table 3:** Chi-square test for the effect of impaction types on cystic lesion

Age	Cystic lesion	Impaction types						Total	χ^2^	*p*
		Vertical	Mesioangular	Horizontal	Distal	Inverted	Buccolingual			
21–25	Yes	0	1	2	0	1	1	5	14.0523	0.1129
	No	7	53	62	1	1	12	136		
26–30	Yes	2	2	4	1	1	1	11	9.355	0.037*
	No	10	52	91	3	3	5	164		
31–35	Yes	0	1	0	0	8	1	10	53.4983	< .0001**
	No	1	30	76	3	7	8	125		
36–40	Yes	0	0	3	0	5	0	8	28.2598	0.0018**
	No	4	20	44	1	4	10	83		
41–45	Yes	1	1	3	0	3	0	8	5.1456	0.2117
	No	2	12	35	0	9	6	64		
46–50	Yes	0	0	1	0	2	1	4	4.7432	0.3398
	No	4	9	21	1	7	5	47		
51–55	Yes	0	1	0	0	2	0	3	7.6882	0.077
	No	2	12	35	0	8	5	62		
56–60	Yes	0	0	0	0	1	0	1	4.9786	0.4878
	No	2	7	21	1	6	3	40		
≥ 61	Yes	0	0	0	0	2	0	2	10.2525	0.1361
	No	2	4	20	1	4	2	33		
Total		37	205	418	12	74	60	806	103.7924	< .0001**

*p < 0.05; **p < 0.01

### Risk factors for adjacent tooth root resorption in patients with LIMTMs

We used logistic regression including age, gender, and impaction type to screen the risk factors for adjacent tooth root resorption in patients with LIMTMs. Using female as the control group, the logistic regression analysis showed no significant difference in the incidence of adjacent tooth root resorption between patients of different genders. We included age as a continuous variable in the analysis, and the results showed that age had an impact on the incidence of adjacent tooth root resorption (odds ratio, 0.971), indicating that the incidence of adjacent tooth root resorption decreased with age. Finally, using horizontal impaction as the control in the analysis of impaction type, we found that the impaction type was not a risk factor for root resorption in teeth adjacent to LIMTMs (Table 4).

**Table 4:** Logistic regression analysis of risk factors for adjacent teeth root resorption

Factor	Standard error	OR	Lower limit of OR	Upper limit of OR	Wald c^2^	*p*
Intercept	0.466	-	-	-	10.9036	0.001
sex(Female as control)	0.261	1.253	0.751	2.089	0.7453	0.388
Age(Continuous variable)	0.0124	0.971	0.948	0.995	5.475	0.0193*
Vertical impaction(Horizontalimpaction as control)	1.0322	0.329	0.044	2.49	1.1585	0.2818
Inverted impaction(Horizontalimpaction as control)	0.4126	1.936	0.862	4.345	2.5622	0.1094
Buccolingual impaction(Horizontalimpaction as control)	0.4695	1.332	0.531	3.342	0.3723	0.5418
Mesioangular impaction(Horizontalimpaction as control)	0.311	1.05	0.571	1.932	0.0245	0.8756
Distal impaction(Horizontalimpaction as control)	1.0652	1.109	0.138	8.948	0.0095	0.9224

* *p* < 0.05;** *p* < 0.01

### Risk factors for LIMTM-related cystic lesions

Logistic regression including the variables age, gender, and type of impaction was applied to screen risk factors for LIMTM-related cystic lesions. Using female as the control group, the logistic regression analysis showed no significant difference in the incidence of LIMTM-related cystic lesions between patients of different genders. We included age as a continuous variable in the analysis and found that age had an impact on the occurrence of LIMTM-related cystic lesions. As age increased, the incidence of LIMTM-related cystic lesions decreased (odds ratio, 0.968). When analyzing the influence of the LIMTM impaction type on cystic lesions, horizontal impaction was used as a control, and the impaction type was found to be a risk factor for LIMTM-related cystic lesions (Table 5).

**Table 5:** Logistic regression analysis of risk factors for cystic lesion

Factor	Standard error	OR	Lower limit of OR	Upper limit of OR	Wald c^2^	*p*
Intercept	0.5887	-	-	-	20.0497	< 0.0001
sex(Female as control)	0.323	1.874	0.995	3.529	3.7783	0.0519
Age(Continuous variable)	0.0147	0.968	0.94	0.996	4.9429	0.0262*
Vertical impaction(Horizontalimpaction as control)	0.6718	3.013	0.808	11.242	2.6957	0.1006
Inverted impaction(Horizontalimpaction as control)	0.4019	19.612	8.922	43.114	54.8409	< 0.0001**
Buccolingual impaction(Horizontalimpaction as control)	0.5931	2.351	0.735	7.518	2.0768	0.1496
Mesioangular impaction(Horizontalimpaction as control)	0.5056	0.919	0.341	2.475	0.028	0.8671
Distal impaction(Horizontalimpaction as control)	1.0902	3.286	0.388	27.844	1.1909	0.2751

* *p* < 0.05;** *p* < 0.01

## Discussion

Low impacted third molars are usually asymptomatic and are often found by X-ray examination. The removal of asymptomatic low impacted third molars is one of the most controversial clinical issues in oral and maxillofacial surgery. The controversy is focused on the fact that some asymptomatic low impacted third molars can lead to adverse consequences, while other asymptomatic impacted third molars may remain clinically silent for life. LIMTMs almost never have a functional role and may increase the risk of cystic formation, caries, and external root resorption of the adjacent second molar [11, 25, 26]. Another frequently proposed reason for the removal of asymptomatic low impacted third molars is to prevent lower incisor crowding [27]. Short-term adverse effects of the removal of LIMTMs include temporary damage to the inferior alveolar nerve and lingual nerve, dry socket, infection, secondary hemorrhage, and restricted mouth opening. Long-term adverse effects of LIMTM extraction are uncommon but can include permanent nerve damage [28]. To avoid these adverse effects, many technical Strategies have been tried, such as Luigi Laino et al. reported a minimally invasive extraoral surgical approach to extract an impacted lower third molar under inferior alveolar canal [29]. A systematic review showed that very few studies have reported LIMTM-related pericoronitis, root resorption, cyst formation, tumor formation, or inflammation/infection [30]. In the present study, we retrospectively analyzed the imaging findings of 806 patients with LIMTMs, focusing on the full life cycle onset characteristics of cystic lesions and adjacent root resorption, and further analyzed the risk factors for cystic lesions and adjacent root resorption caused by LIMTMs.

The results showed that the overall prevalence of adjacent tooth root resorption caused by LIMTMs was 8.81%, which was lower than in previous studies [31–33]. This may have occurred because this study focused on LIMTMs (full bony impaction), whereas previous studies focused on soft tissue-impacted third molars. Additionally, the age span of the patients included in this study was large (21–80 years), and the prevalence rate of patients aged > 45 years decreased sharply; this reduced the overall prevalence rate to a certain extent. The prevalence of adjacent tooth root resorption showed a certain correlation with age. As age increased, the incidence of root resorption in adjacent teeth generally exhibited a trend of first increasing and then decreasing, reaching its peak at the age of 41 to 45 years and then sharply decreasing. The chi-square test and logistic regression analysis showed that age was an important factor affecting the incidence rate of root resorption of teeth adjacent to LIMTMs, while the type of LIMTM impaction was a pathogenic factor for root resorption only in the 21- to 25-year age group. This suggests that LIMTMs are in a more active state before the age of 45 years and can thus be removed in patients in this age group. LIMTMs are usually in a resting state after the age of 45 years and therefore have a lower likelihood of causing adjacent tooth root resorption. If the surgical risk is high, conservative treatment with regular follow-up can be implemented. Nunn et al. [2] retrospectively analyzed the influence of impacted third molars on adjacent second molars in 416 men and found that asymptomatic third molars with bone impaction increased the likelihood of lesion development in adjacent second molars.

Cystic disease is a serious complication of LIMTMs. Cysts are usually asymptomatic during their development and progression and are often found by X-ray examination performed for other purposes. A systematic review showed that very few studies have focused on LIMTM-related cyst and tumor formation [30]. In the present study, 51 cystic lesions occurred among 806 patients with LIMTMs (prevalence rate of 6.33%). The prevalence of cystic lesions showed a trend of first increasing and then decreasing, and the prevalence peaked at the age of 41 to 45 years. The logistic regression analysis results showed that age and inverted impaction type were risk factors for LIMTM-related cystic lesions. The incidence rate of cystic lesions gradually decreased with age, and the incidence rate of cystic lesions caused by inverted impacted LIMTMs was higher than that of other types. The chi-square analysis results showed that the effect of the type of LIMTM impaction on the occurrence of cystic lesions was statistically significant, especially in the age groups of 26 to 30, 31 to 35, and 36 to 40 years. These results suggest a preference for surgical removal of LIMTMs, especially inverted impacted LIMTMs, in patients aged < 40 years. LIMTMs in patients aged > 45 years may be treated conservatively with regular follow-up, but surgical removal is still recommended for inverted impacted LIMTMs to avoid cyst formation.

However, some limitations exit in the study. On the one hand, root resorption can be divided into different degrees according to the spread range. In this study, root resorption of adjacent teeth was not classified in detail. On the other hand, LIMTMs associated cystic lesions lack a clear pathological classification. In the next study, the risk factors related to the degree of root resorption in adjacent teeth of LIMTMs and pathological classification of LIMTMs related cystic lesions should be explored in detail.

## Conclusions

Within the limitations of this study, age was found to be an independent risk factor for adjacent tooth root resorption in patients with LIMTMs. Moreover, age and LIMTM impaction type were found to be independent risk factors for LIMTM-related cystic lesions. Therefore, the full life cycle management strategy for LIMTMs may need to be individualized. Treatment strategies for surgical removal are recommended for LIMTMs in patients younger than 41 to 45 years, especially for inverted, mesioangular, and horizontally impacted LIMTMs. LIMTMs in patients older than 41 to 45 years may be treated conservatively with regular follow-up, but surgical removal of inverted impacted LIMTMs is still recommended to avoid cyst formation.

## Data Availability

All relevant datasets and their supporting information files generated and/or analyzed during this study are available from the corresponding author upon reasonable request.

## Abbreviations

LIMTMs: Low impacted mandibular third molars
CBCT: Cone-beam computed tomography
